# Describing the health-related quality of life of Māori adults in Aotearoa me Te Waipounamu (New Zealand)

**DOI:** 10.1007/s11136-023-03399-w

**Published:** 2023-03-16

**Authors:** Trudy Sullivan, Georgia McCarty, Emma Wyeth, Robin M. Turner, Sarah Derrett

**Affiliations:** 1 Department of Preventive and Social Medicine, University of Otago, 18 Frederick Street, Dunedin, 9016 New Zealand; 2 Ngāi Tahu Māori Health Research Unit, Division of Health Sciences, University of Otago, Dunedin, New Zealand; 3 Biostatistics Centre, Division of Health Sciences, University of Otago, Dunedin, New Zealand

**Keywords:** Māori, Health-related quality of life, EQ-5D-5L, Indigenous, Preferences, Aotearoa me Te Waipounamu New Zealand

## Abstract

**Purpose:**

In Aotearoa me Te Waipounamu (New Zealand; NZ) there are considerable inequities in health status and outcomes for Māori, the Indigenous peoples of NZ. It is therefore important that the health status and preferences of Māori are specifically considered in healthcare policy and decision making. This paper describes the health-related quality of life of 390 Māori adults who took part in the NZ EQ-5D-5L valuation study.

**Methods:**

Responses on the five dimensions of the EQ-5D-5L were dichotomised into “no problems” and “any problems”, summarised and disaggregated by age group. Mean preference weights were reported by age group and overall. Mean utility values (calculated by applying each participant’s preference weights to their EQ-5D-5L profile) were summed and respective means and standard deviations reported by age, chronic disease status and disability.

**Results:**

The EQ-5D-5L dimensions with the highest proportion of participants reporting any problems were pain/discomfort (61.5%) and anxiety/depression (50%). The most commonly-reported chronic disease was mental illness/distress (24.6%). Anxiety/depression ranked as the most important dimension, with usual activities, the least important. The mean utility value was 0.83 with the lowest value (0.79) found in the 18–24 and 45–54 age groups. For participants with at least one chronic disease the mean utility value was 0.76 compared to 0.91 for those with none.

**Conclusion:**

To reduce inequities experienced by Māori it is crucial that the health status of Māori and the values Māori place on health-related quality of life are properly understood. This can only be achieved using Māori-specific data.

## Plain English Summary

In Aotearoa me Te Waipounamu (New Zealand; NZ) there are considerable inequities in health status and outcomes for Māori, the Indigenous peoples of NZ. To reduce inequities experienced by Māori it is crucial that the health status of Māori and the values Māori place on health-related quality of life are properly understood. This can only be achieved using Māori-specific data. This is the first study to report Māori population norms and health preferences for the EQ-5D-5L. Almost a third of the study sample had two or more chronic diseases. Mental illness/distress was the most common condition reported, with the youngest age groups notably affected by anxiety and depression. These results indicate the importance of using Māori-specific data to inform policy development and economic decision making in NZ.

## Introduction

The Waitangi Tribunal is a permanent commission of inquiry in Aotearoa me Te Waipounamu (New Zealand; NZ) that investigates and makes recommendations on claims brought by Māori (the Indigenous peoples of NZ) relating to alleged breaches of Te Tiriti o Waitangi (the Treaty of Waitangi) made by the Crown [1]). Te Tiriti o Waitangi (signed in 1840) is an agreement between Māori and non-Māori which includes a pledge for equality between Māori and non-Māori [2]. In 2019, the Waitangi Tribunal released a report on Stage One of the Health Services and Outcomes Kaupapa Inquiry: WAI2575 [3]. The report concluded that, as per Te Tiriti o Waitangi, the primary healthcare system has failed to achieve equitable health outcomes for Māori [3, 4].

Considerable inequities in health status and outcomes for Māori are well-documented, with non-Māori experiencing lower incidence, prevalence, and mortality from chronic disease [4–6]. In 2017–2019, life expectancy at birth for non-Māori males was 80.9 (relative to 73.4 years for Māori males) and 84.4 for non-Māori females (relative to 77.1 years for Māori females) [7]. The most recent Ministry of Health statistics report that the cardiovascular disease mortality rate for Māori was twice as high compared to non-Māori, diabetes prevalence was almost double, younger Māori (5–34 years) were almost twice as likely to be hospitalised for asthma, and Māori aged ≥ 25 years had significantly higher cancer registration rates with cancer mortality more than 1.5 times higher for Māori [8]. Many policy and systemic factors contribute to such inequities [6, 9].

The dominant narrative of individual blame to explain such inequities for Māori, including poor health behaviours and lifestyle choices [10], fails to acknowledge the history of colonisation and failures of the healthcare system. For example, Māori are less likely to be screened for cardiovascular disease [11] and prostate cancer [12]; to visit a general practitioner (and appointment times are shorter) [11]; and to receive life-preserving surgical interventions than non-Māori [13]. As well as evidence of differential access to healthcare services, there are also concerns about the quality of care provided to Māori [11, 12].

The link between colonisation and ongoing health inequities in NZ is also well documented [14–16] and spans multiple generations [17]. There is evidence that structural discrimination within the healthcare system is responsible for reduced access to, and effectiveness of, health services and interventions for Māori, including those related to injury [4, 18–22]. To effectively address these issues and reduce the inequities in health outcomes for Māori, it is important that health and outcomes of Māori are properly understood.

A common way to describe or measure health status is to use health-related quality of life (HRQoL) measures (i.e. structured questionnaires) either independently or alongside other outcome measures, e.g. mortality, hospital admissions, and/or clinical parameters [23]. These measures typically include physical, mental, emotional and social components and can be used to understand the burden of disease, identify health inequities, inform epidemiological studies, evaluate healthcare interventions and treatments, undertake health technology assessments and inform decisions on how to allocate health resources [24]. Internationally, the HRQoL measure most commonly used, including in NZ, is the five-dimension EQ-5D, developed by the EuroQol Group in 1990 [25, 26]. The original version, the EQ-5D-3L [27], has three levels of severity ranging from “no problems” to “confined/unable to do/extreme problems” on each of the five dimensions—mobility, self-care, usual activities, pain/discomfort and anxiety/depression. The five-level version, created in 2009, the EQ-5D-5L [28], has five levels of severity ranging from “no problems” to “unable to do/extreme problems”. Since 1999, NZ researchers, funders and decision-makers (including Pharmac, NZ’s national medicine and related product buying agency [29]) have been using the EQ-5D-3L measure and social value set (which comprises values for each of the 243 possible health states) [30]. In 2018, a social value set for the EQ-5D-5L (comprising 3125 health state values) was created for NZ by some of the present authors using the personal value sets of 2468 participants [31]. The sample for the earlier NZ EQ-5D-3L population survey included 7.6% (n = 70) Māori; the 2018 NZ EQ-5D-5L sample included 15.8% (n = 390) Māori, and therefore is more closely aligned with the population (i.e. 14.9% of NZ adults identified as Māori in the 2013 NZ Census [32]).

Though the NZ EQ-5D-5L value set may be representative in terms of the proportion of the total population who completed the survey, the EQ-5D-5L instrument does not encompass a holistic Māori view of health which acknowledges wider social, cultural and economic determinants, focussing on collective, rather than individual health [6, 33]. For example, a well-known Māori model of health, Te Whare Tapa Whā [34], describes hauora (overall health and wellbeing) as a whare (house), with four equally strong walls, representing four core dimensions: tinana (body), whānau (family), wairua (spirit), and hinengaro (mental). Given the EQ-5D was not developed with these factors in mind, instead drawing on popular Western conceptions of health, it is unlikely that it captures all aspects of health important to Māori. Considering the HRQoL of Māori, including capturing aspects of health important to Māori, is an important obligation of the NZ Government aligned with Te Tiriti o Waitangi [2]. The Waitangi Tribunal confirmed in the WAI2575 report that the Crown has failed to produce equitable health outcomes for Māori: “The Tribunal found that the Crown has systematically contravened obligations under Te Tiriti across the health sector.” [36, p. 209]. To achieve equity of health outcomes, the health status and preferences of Māori need to be better understood to ensure Māori needs and aspirations are prioritised and for healthcare resources to be appropriately allocated to improve outcomes and reduce inequities.

The aim of this paper is to describe the HRQoL of 390 Māori adults who took part in the NZ EQ-5D-5L valuation study [31].

## Methods

### Data

Māori data from the 2018 NZ EQ-5D-5L valuation study were used for the analysis [31, 36]. As described elsewhere, an international research company was contracted to recruit a representative sample of the NZ population in terms of age, gender, ethnicity and geographic location [31]. Of the 2468 participants aged ≥ 18 years at the time of the survey, 390 identified as Māori as measured by the NZ Census question about ethnicity [37]. Ethics approval for the NZ EQ-5D-5L study and associated analyses was approved by the University of Otago Human Ethics Committee (D17/297).

### Variables

As mentioned, the EQ-5D-5L describes health on five dimensions—mobility, self-care, usual activities, pain/discomfort and anxiety/depression—with five levels of severity on each dimension (i.e. no/slight/moderate/severe/extreme problems). The variables used in this study included each participant’s EQ-5D-5L profile, EQ-VAS score, preference weights and utility value as described below:

1. *EQ-5D-5L profile:* a 5-digit profile representing a participant’s health status according to the severity of problems reported for each dimension of the EQ-5D-5L, e.g. where 1 = no problems, and 5 = extreme problems, an individual’s overall profile across the five dimensions could be presented as 12342.
2. *EQ-VAS score:* a number between 0 and 100, with 0 indicating the “worst health” and 100 the “best health” participants can imagine.
3. *EQ-5D-5L preference weights and utility value:* preference weights reflect the relative importance of each of the dimensions (and levels). Each participant’s preference weights were applied to their EQ-5D-5L profile to obtain a utility value where 1 equates to perfect health and 0 to being dead (with values less than 0 equating to states perceived to be worse than dead).

Sociodemographic information collected directly from participants included gender, age, ethnicity, education level, employment status, individual income, living arrangements (alone or with others), region of residence, long-term disability (i.e. a condition that prevents a person from doing everyday things that other people can do, lasting ≥ 6 months), and chronic disease or illness (i.e. a physical or mental illness diagnosed by a doctor, expected to last for ≥ 6 months).

### Statistical analysis

Participants’ EQ-5D-5L profiles were categorised into two groups—“no problems” and “any problems” which included “slight, moderate, severe or extreme problems”—and reported as numbers and percentages by age group. The EQ-VAS scores of participants were summed, and their respective means and standard deviations calculated to produce mean EQ-VAS scores for the full sample and by age group.

Mean preference weights were calculated for the five EQ-5D dimensions and disaggregated by age group. Utility values were summed across all participants and the respective means and standard deviations calculated by age group and overall.

To explore chronic disease status in more detail (both in terms of describing HRQoL and its effect on utility), responses on the 22 chronic disease or illness categories were grouped into: (1) chronic disease status (yes/no); (2) number of chronic diseases (ranging from 0 to 6+); and (3) eight chronic disease groups based on ICD-10 classifications [38]. Mean utility values and standard deviations were calculated for each of these groupings and by disability status.

Statistical analyses were performed using STATE/SE 16.1 [39].

## Results

### Participants

The sociodemographic characteristics of the 390 Māori participants are reported in Table 1. Where available, data for the general Māori population according to the 2013 Census information are included for comparative purposes. Of the 390 participants, 63.6% were female, 24.4% were aged 35–44 years, 53.6% reported no or secondary school qualifications, 50% were employed (in either part- or full-time work), 72.8% had a personal income of ≤ $50,000, and 32.3% had a long-term disability lasting 6 months or more.

**Table 1:** Sociodemographic characteristics of Māori participants (n = 390) and 2013 New Zealand Census data (where available)

Characteristic	Participants		NZ census data
	*n *= 390	%	%
Gender			
Male	141	36.2	46.3
Female	248	63.6	53.7
Age (years)			
18**–**24	56	14.4	19.9
25**–**34	92	23.6	19.8
35**–**44	95	24.4	20.4
45**–**54	64	16.4	18.8
55**–**64	50	12.8	12.3
≥ 65	33	8.5	8.9
Education level^a^			
No qualifications/Secondary school	209	53.6	67.9
Other post-secondary school qualifications	83	21.3	13.6
University degree or equivalent	98	25.1	9.1
Employment status			
Full-time work (≥ 30 h per week)	143	36.7^b^	
Part-time work (< 30 h per week)	52	13.3	
Not in paid work (including people on a benefit)	84	21.5	
Student/homemaker	75	19.2	
Retired	27	6.9	
Others (including self-employed)	9	2.3	
Individual income			
≤ $20,000	112	28.7	42.5
$20,001**–**$30,000	80	20.5	13.1
$30,001**–**$50,000	92	23.6	19.6
$50,001**–**$70,000	57	14.6	9.7
$70,001**–**$100,000	36	9.2	4.6
≥ $100,001	13	3.3	2.3
Live alone			
Yes	32	8.2	9.0
No	358	91.8	91.0
Region of residence^a^			
Northern	126	32.3	31.4
Midlands	114	29.2	31.8
Central	98	25.1	22.8
Southern	52	13.3	13.9
Long-term disability (lasting ≥ 6 months)			
No	264	67.7	74
Yes	126	32.3	26
Chronic disease			
No	170	43.6	
Yes	220	56.4	

^a^NZ population statistics include people ≥ 15 years

^b^2017 September quarter employment and unemployment rates were 63.5% and 9.9%

The sample is reasonably representative of the Māori population. However, males, those aged 18–24 years, and participants reporting income ≤ $20,000 were under-represented in the sample, while participants with post-secondary school qualifications or higher were over-represented. There were also more participants with a long-term disability compared to the general Māori population.

### Health status of participants

The self-reported health status of participants, as described on the EQ-5D-5L and VAS, are presented by age group in Table 2. For the full sample, the dimension with the highest proportion of participants reporting problems was Pain/Discomfort (61.5%, n = 240; comprised of 162, 62, 14 and 2 with slight, moderate, severe and extreme problems respectively), followed by Anxiety/Depression (50.0%, n = 195; comprised of 108, 61, 14 and 12 with slight, moderate, severe and extreme problems respectively), Usual Activities (28.5%, n = 111; comprised of 76, 24 and 8 with slight, moderate and severe problems respectively and 3 ‘unable to’), Mobility (27.7%, n = 108; comprised of 68, 33 and 4 with slight, moderate and severe problems respectively and 3 ‘unable to’) and Self-care (9.5%, n = 37; comprised of 28, 6 and 2 with slight, moderate and severe problems respectively and 1 ‘unable to’).

**Table 2:** HRQoL by EQ-5D-5L dimension and EQ-VAS score, disaggregated by age with column percentages shown

Dimension	Age group (years)						
	18–24	25–34	35–44	45–54	55–64	≥ 65	Total
	n (%)	n (%)	n (%)	n (%)	n (%)	n (%)	n (%)
Mobility							
No problems	46 (82.1)	77 (83.7)	73 (76.8)	40 (62.5)	29 (58.0)	17 (51.5)	282 (72.3)
Any problems	10 (17.9)	15 (16.3)	22 (23.2)	24 (37.5)	21 (42.0)	16 (48.5)	108 (27.7)
Self-care							
No problems	54 (96.4)	81 (88.0)	87 (91.6)	53 (82.8)	46 (92.0)	32 (97.0)	353 (90.5)
Any problems	2 (3.6)	11 (12.0)	8 (8.4)	11 (17.2)	4 (8.0)	1 (3.0)	37 (9.5)
Usual activities							
No problems	40 (71.4)	75 (81.5)	68 (71.6)	39 (60.9)	32 (64.0)	25 (75.8)	279 (71.5)
Any problems	16 (28.6)	17 (18.5)	27 (28.4)	25 (39.1)	18 (36.0)	8 (24.2)	111 (28.5)
Pain/discomfort							
No problems	29 (51.8)	42 (45.7)	38 (40.0)	17 (26.6)	14 (28.0)	10 (30.3)	150 (38.5)
Any problems	27 (48.2)	50 (54.3)	57 (60.0)	47 (73.4)	36 (72.0)	23 (69.7)	240 (61.5)
Anxiety/depression							
No problems	20 (35.7)	27 (29.3)	50 (52.6)	37 (57.8)	34 (68.0)	27 (81.8)	195 (50.0)
Any problems	36 (64.3)	65 (70.7)	45 (47.4)	27 (42.2)	16 (32.0)	6 (18.2)	195 (50.0)
Total	56 (14.4)	92 (23.6)	95 (24.4)	64 (16.4)	50 (12.8)	33 (8.5)	390 (100.0)
EQ VAS score							
Mean	71.2	70.2	68.8	70.2	76.3	80.3	71.6
Standard deviation	19.9	18.6	19.4	20.1	20.0	15.3	19.4

The proportion of participants reporting problems with mobility and pain/discomfort generally increased with age. Those aged 25–34 years had the lowest proportion (16.3%) of participants experiencing mobility problems; those aged ≥ 65 years had the highest proportion (48.5%). Problems with pain/discomfort were consistently high across all age groups, ranging from 48.2% for those aged 18–24 years to 73.4% for those aged 45–54 years.

The highest proportion of participants experiencing anxiety/depression were younger with 64.3% of 18–24 year-olds and 70.7% of 25–34 year-olds, reporting problems. For those aged ≥ 65 years, the proportion (18.2%) was much lower. Problems with usual activities ranged from 18.5% for those aged 25–34 years to 39.1% for those aged 45–54 years. Problems with self-care were lower compared to other dimensions with participants aged 45–54 years reporting the highest proportion of problems (17.2%) and participants aged ≥ 65 years, the lowest (3%).

The mean EQ-VAS score for the total sample was 71.6. Participants aged ≥ 65 years had the highest EQ-VAS score (80.3) while participants aged 35–44 years had the lowest (68.8).

### Mean preference weights and utility values

The mean preference weights for the EQ-5D-5L dimensions, overall and by age, are presented in Table 3. The mean weights are normalised so that the best health state possible equals one [i.e. the level 1 values (in bold) add to 1]. The dimension considered most important overall (i.e. valued the most, in terms of *not* experiencing it) was Anxiety/Depression (0.211), followed by Self-care (0.209), Pain/Discomfort (0.199), and Mobility (0.198). The dimension considered least important was Usual Activities (0.184).

**Table 3:** Mean preference weights and utility values by age group and overall

EQ-5D-5L dimensions	Mean preference weights by age group and overall						
	18–24	25–34	35–44	45–54	55–64	≥ 65	Mean
Mobility							
I have no problems walking about	**0.202**	**0.199**	**0.194**	**0.191**	**0.201**	**0.206**	**0.198**
I have slight problems walking about	0.166	0.163	0.16	0.16	0.162	0.179	0.163
I have moderate problems walking about	0.123	0.119	0.12	0.121	0.118	0.141	0.122
I have severe problems walking about	0.067	0.064	0.066	0.067	0.064	0.08	0.067
I am unable to walk about	0	0	0	0	0	0	0
Self-care							
I have no problems washing or dressing myself	**0.206**	**0.207**	**0.215**	**0.198**	**0.219**	**0.205**	**0.209**
I have slight problems washing or dressing myself	0.165	0.167	0.171	0.162	0.175	0.177	0.169
I have moderate problems washing or dressing myself	0.118	0.121	0.123	0.118	0.125	0.137	0.122
I have severe problems washing or dressing myself	0.062	0.064	0.065	0.063	0.066	0.076	0.065
I am unable to wash or dress myself	0	0	0	0	0	0	0
Usual activities							
I have no problems doing my usual activities	**0.201**	**0.180**	**0.177**	**0.197**	**0.182**	**0.159**	**0.184**
I have slight problems doing my usual activities	0.169	0.151	0.145	0.164	0.154	0.136	0.153
I have moderate problems doing my usual activities	0.128	0.114	0.107	0.123	0.117	0.103	0.115
I have severe problems doing my usual activities	0.07	0.062	0.058	0.067	0.065	0.057	0.063
I am unable to do my usual activities	0	0	0	0	0	0	0
Pain/discomfort							
I have no pain or discomfort	**0.185**	**0.202**	**0.207**	**0.210**	**0.183**	**0.198**	**0.199**
I have slight pain or discomfort	0.149	0.168	0.176	0.177	0.148	0.17	0.166
I have moderate pain or discomfort	0.107	0.126	0.135	0.133	0.107	0.131	0.125
I have severe pain or discomfort	0.057	0.069	0.075	0.072	0.057	0.073	0.068
I have extreme pain or discomfort	0	0	0	0	0	0	0
Anxiety/depression							
I am not anxious or depressed	**0.206**	**0.213**	**0.207**	**0.203**	**0.215**	**0.231**	**0.211**
I am slightly anxious or depressed	0.161	0.171	0.166	0.163	0.169	0.191	0.168
I am moderately anxious or depressed	0.113	0.123	0.12	0.117	0.12	0.142	0.121
I am severely anxious or depressed	0.059	0.066	0.063	0.062	0.063	0.076	0.064
I am extremely anxious or depressed	0	0	0	0	0	0	0
Utility value							
Mean	0.79	0.83	0.82	0.79	0.86	0.88	0.83
Standard deviation	0.41	0.21	0.26	0.26	0.18	0.16	0.26

When disaggregated by age group, the highest weights for Mobility (0.206) and Anxiety/Depression (0.231) were in the ≥ 65 age group. The highest weight (0.201) for Usual Activities was in the 18–24 age group, while the lowest weight (0.159) was in the ≥ 65 age group. For Pain/Discomfort, the lowest weights (0.183 and 0.185) were in the 55–64 and 18–24 age groups respectively, with Pain/Discomfort being the least important dimension for those aged 18–24 years. In contrast, Pain/Discomfort was the most important dimension (0.210) for participants aged 45–54 years and the second most important (0.207) for those aged 35–44 years.

The mean utility value was 0.83 (SD 0.26) with the highest utility value (0.86, SD 0.18) in the 55–64 age group, and the lowest utility values (0.79, SD 0.41; 0.79, SD 0.26) in the 18–24 and 45–54 age groups, respectively.

### Chronic disease status

As reported in Table 4, over half of the sample had at least one chronic disease (n = 220; 56.4%) with 32.8% of participants reporting more than one. When grouped by condition, the most common chronic diseases were mental illness/distress (24.6%), musculoskeletal (18.2%), respiratory (17.2%) and diabetes (10.5%).

**Table 4:** Chronic disease and disability status with mean utility values (n = 390)

Chronic disease status (n = 390)	No	%	Mean	Utility
				SD
Chronic disease				
No	170	(43.6)	0.91	0.13
Yes	220	(56.4)	0.76	0.31
Number of chronic diseases/conditions				
0	170	(43.6)	0.91	0.13
1	92	(23.6)	0.88	0.13
2	57	(14.6)	0.73	0.41
3	37	(9.5)	0.67	0.33
4	18	(4.6)	0.70	0.32
5	5	(1.3)	0.59	0.21
6 or more	11	(2.8)	0.70	0.32
Chronic disease groups (by ICD-10 code)				
Mental illness/distress	96	(24.6)	0.65	0.40
Musculoskeletal	71	(18.2)	0.70	0.30
Respiratory	67	(17.2)	0.73	0.29
Diabetes	41	(10.5)	0.79	0.23
Neurological	36	(9.2)	0.66	0.33
Cardiovascular	31	(7.9)	0.78	0.26
Bowel and digestive	19	(4.9)	0.64	0.35
Cancer	9	(2.3)	0.91	0.14
Long-term disability				
No	264	(67.7)	0.89	0.15
Yes	126	(32.3)	0.69	0.37

The mean utility value for participants with at least one chronic disease was lower [0.76 (SD 0.31)] compared to participants without a chronic disease [0.91 (SD 0.13)]. Generally, as the number of chronic diseases increased, mean utility values decreased, with participants reporting five conditions having the lowest mean utility value of 0.59 (SD 0.21). In terms of disease groupings, the highest mean utility value was in the cancer group (0.91, SD 0.14), and the lowest values were in the bowel and digestive (0.64, SD 0.35), mental illness/distress (0.65, SD 0.40) and neurological (0.66, SD 0.30) groups.

Mean utility values were lower for people with a long-term disability (0.69, SD 0.37) compared to those without (0.89, SD 0.15).

## Discussion

Like many Indigenous populations worldwide, Māori experience disproportionate burdens of disease, disability, mortality, and morbidity compared to non-Māori [4, 11, 14, 15, 40–44]. The extent of inequities provides clear evidence of the failure within the healthcare system to deliver equitable health to Māori. The WAI2575 claim [3] reported that the primary healthcare system has failed to provide services that achieve equitable outcomes for Māori. The consequences are increasing rates of chronic disease, comorbidity and poorer health outcomes, leading to greater healthcare costs and growing inequities [6, 8, 10, 11, 40, 41]. If the NZ Government is to meet its Treaty obligations [1], equitable health outcomes for Māori must be attained. As part of achieving this, the needs and health preferences of Māori need to be specifically considered.

Despite the widespread use of the EQ-5D in decision making in NZ [29, 45–47], Māori-specific preferences are not considered. With the creation of a NZ EQ-5D-5L social value set comprised of personal (i.e. individual) value sets, this is now possible. This study is the first to investigate Māori EQ-5D-5L general population norms and health preferences. Understanding Māori-specific preferences in health is an important first step in meeting the obligations of the Treaty of Waitangi [1] and the United Nations Declaration on the Rights of Indigenous Peoples (UNDRIP) [48].

The participants (n = 390) in this study were generally representative of the Māori population though there were proportionately fewer males and young people (18–24 years). Almost two thirds (61.5%) of the study sample reported having problems with pain/discomfort and 50% with anxiety/depression. Considerably fewer (9.5%) participants had problems with self-care. The mean EQ-5D-5L utility value was 0.83 (out of 1), comparatively higher than the mean EQ-VAS score of 71.6 (out of 100). Although related, utility values and EQ-VAS scores represent health status differently. For instance, a participant’s utility value is based on their health status as reported on the five discrete dimensions of the EQ-5D. In contrast, when a participant scores their health on the EQ-VAS, they may think about many different aspects of health resulting in a EQ-VAS score lower than their utility value. Another possibility for the difference between utility values and EQ-VAS scores is that the EQ-5D does not capture all components of health important, or relevant, to Māori, given the holistic and inter-connected nature in which Māori view health (discussed further below).

Over half of the sample had at least one chronic disease (56.4%), with 32.8% having two or more. Having a long-term disability or chronic disease negatively impacted HRQoL. The conditions having the most impact on utility were bowel and digestive conditions, mental illness/distress and neurological conditions. Generally, as the number of chronic conditions increased, mean utility decreased (i.e. worsened). Māori are disproportionately affected by chronic disease, including mental health conditions, experiencing a higher burden of anxiety, depression and mental distress compared to non-Māori [49, 50]. In this study, up to 71% of the participants aged 18–34 years reported problems with anxiety/depression. In terms of health preferences, the dimension considered the most important overall (i.e. the dimension participants wanted to avoid the most) and therefore the one having the largest impact on utility, was anxiety/depression.

With the many and varied sustained inequities borne by Māori and the ongoing impacts of these,[4–8, 11–18, 22] unless a more concerted effort is made to inform health decisions, these inequities and impacts will continue. Determining who is most affected by poor health, the contributing factors and the aspects of health considered most important to people is essential for informed decision-making (explained below).

The NZ EQ-5D-3L value set has been used for cost utility analysis and by extension, resource allocation, since 1999. With the creation of an EQ-5D-5L value set, not only can the personal value sets that comprise the social value set be used to inform decision making, the HRQoL of Māori (i.e. the population norms as described in this study) can be used to identify areas of priority. Using Māori-specific HRQoL data enable a non-deficit approach to be implemented by focusing on Māori, in contrast to using general population norms or a comparative (deficit) approach. The data can be used by researchers to inform their work and to compare findings. Understanding the health status of subgroups of the Māori population will assist health practitioners and policy makers in identifying potential areas of need. For example, only 35.7% of participants aged 18–24 years and 29.3% of participants aged 25–34 years indicated they had no problems with anxiety/depression. Health agencies such as Te Aka Whai Ora (Māori Health Authority) could use this type of information in their health planning and promotion.

The use of mean population weights (i.e. the only option with the NZ EQ-5D-3L) in cost utility analysis could potentially hide the true effect. Using the weights of the subgroup of interest (e.g. young Māori) would determine the impact on that specific group. For instance, using Māori-specific preference weights in a cost utility analysis for a condition that disproportionately affects Māori such as acute respiratory illness could contribute to more informed healthcare decisions, and potentially better health outcomes for Māori.

However, while this is a strength of using the NZ EQ-5D-5L, the EQ-5D (or any existing HRQoL measure) may not encompass all aspects of health important to Māori. Māori value dimensions of health that go beyond Western concepts that are often included in HRQoL measures, such as wairua (spirit), te taiao (the environment), te ao Māori (the Māori world), and whānau (family) [21, 34, 51–55]. We are currently exploring whether any existing HRQoL measures adequately capture the components of health important to rangatahi Māori (Māori youth) or whether any existing measure(s) need to be adapted or a bespoke measure created [56]. Future research will also focus on adult HRQoL measures and the appropriateness to Māori of using choice-based methods to elicit health state preferences. In the meantime, it is important that HRQoL of Māori is specifically considered, something that is achievable with the EQ-5D.

There are potential limitations relating to the source data used in this study. Though the Māori population is adequately represented in terms of the percentage of the NZ population who identify as Māori, selection bias is possible in regard to who is more likely to complete online surveys (e.g. computer and internet access; time and confidence to complete the survey). In this sample, Māori participants with higher levels of education were over-represented, and those earning ≤ $20,000 were under-represented. As higher levels of income and education are known to have positive effects on utility, the mean EQ-VAS scores and utility values in this study may be higher than expected with a more representative sample [57–59].

## Conclusion

To address current health inequities, the health status and preferences of Māori need to be specifically and appropriately understood to ensure equitable health outcomes for Māori. This study is the first to report Māori population norms and health preferences for the EQ-5D-5L. Similar to national statistics, almost a third of the study sample had two or more chronic diseases. Mental illness/distress was the most common condition reported, with the youngest age groups notably affected by anxiety and depression. These results indicate the importance of using Māori-specific data to inform policy development and economic decision making in NZ.

## Data Availability

All data generated or analysed for this Māori-specific study are included in this published article.
